# Anti-Obesity Effects of a *Prunus persica* and *Nelumbo nucifera* Mixture in Mice Fed a High-Fat Diet

**DOI:** 10.3390/nu12113392

**Published:** 2020-11-04

**Authors:** Jungbin Song, Jiye Kim, Hyo Jin Park, Hocheol Kim

**Affiliations:** 1 Department of Herbal Pharmacology, College of Korean Medicine, Kyung Hee University, 26 Kyungheedae-ro, Dongdaemun-gu, Seoul 02447, Korea; jbsong@khu.ac.kr; 2 Department of Herbal Pharmacology, College of Korean Medicine, Graduate School, Kyung Hee University, 26 Kyungheedae-ro, Dongdaemun-gu, Seoul 02447, Korea; briskia@naver.com; 3 Korea Institute of Science and Technology for Eastern Medicine (KISTEM), NeuMed Inc., 88 Imun-ro, Dongdaemun-gu, Seoul 02440, Korea; rnfyddyd@neumed.co.kr

**Keywords:** *Prunus persica*, *Nelumbo nucifera*, high-fat diet, adipokine, fatty acid oxidation, lipid synthesis, obesity

## Abstract

*Prunus persica* and *Nelumbo nucifera* are major crops cultivated worldwide. In East Asia, both *P. persica* flowers and *N. nucifera* leaves are traditionally used for therapeutic purposes and consumed as teas for weight loss. Herein, we investigated the anti-obesity effects of an herbal extract mixture of *P. persica* and *N. nucifera* (HT077) and the underlying mechanism using a high-fat diet (HFD)-induced obesity model. Male C57BL/6 mice were fed a normal diet, HFD, HFD containing 0.02% orlistat (positive control), or HFD containing 0.1, 0.2, or 0.4% HT077 for 12 weeks. HT077 significantly reduced final body weights, weight gain, abdominal fat weights, liver weights, and hepatic levels of triglycerides and total cholesterol. HT077 also lowered glucose, cholesterol, alanine aminotransferase (ALT), aspartate aminotransferase (AST), and leptin levels and increased AST/ALT and adiponectin/leptin ratios and adiponectin levels. Real-time polymerase chain reaction analysis showed that HT077 decreased the expression of lipogenic genes and increased the expression of fatty acid oxidation-related genes in adipose tissue. Our results indicate that HT077 exerts anti-obesity effects and prevents the development of obesity-related metabolic disorders. These beneficial effects might be partially attributed to ameliorating adipokine imbalances and regulating lipid synthesis and fatty acid oxidation in adipose tissue.

## 1. Introduction

The steady increase in overweight and obese individuals has become a serious health problem worldwide. Currently, it is estimated that nearly one-third of the world’s population is overweight or obese [1]. Obese people are at a high risk for many serious diseases, including diabetes, cardiovascular diseases, nonalcoholic fatty liver disease (NAFLD), osteoarthritis, and cancers [2]. Obesity and related diseases not only damage a person’s health and quality of life but also have become a socioeconomic burden. For the management of such conditions, lifestyle interventions, including a low-calorie diet, physical activity, and behavior therapy, are ideal, but motivation and adherence are not easy [3]. Pharmacological interventions for long-term use include orlistat, lorcaserin, liraglutide, phentermine-topiramate, and naltrexone-bupropion. These drugs are approved for moderately or severely obese patients with a body mass index (BMI) ≥30 or BMI ≥27 with comorbidities and have a high incidence of side effects [4]. Considering the limitations of conventional medication and the complex nature of the pathogenesis of obesity, more safe and effective novel approaches are required.

Natural herbal products with anti-obesity effects have attracted much attention from researchers [5]. In East Asia, many traditional medicinal herbs are edible, and diet therapy using synergistic polyherbal formulations has been well accepted for a long time [6]. Given that the simultaneous modulation of multiple targets is required to manage multifactor diseases such as obesity [7], the use of synergistic polyherbal formulations will act on multiple targets, enhancing their anti-obesity effects. The introduction of a combination of edible herbs from traditional Asian medicine as dietary herbal therapy could be a promising approach for the treatment of obesity and associated disorders.

From these perspectives, we screened the anti-obesity effects of edible medicinal herbs used for weight and fat loss in East Asia. Through multiple screening processes using a high-fat diet (HFD)-induced obesity model, we revealed for the first time, to our knowledge, that the flowers of *Prunus persica* (L.) Batsch (family Rosaceae) effectively suppress abdominal fat accumulation and hyperglycemia in obese mice by reducing lipogenesis and increasing fatty acid oxidation in the liver [8]. Interestingly, *P. persica* has been shown more effective in reducing adiposity and associated hyperglycemia when combined with the leaves of *Nelumbo nucifera* Gaertn. (family Nelumbonaceae) in obese mice [9].

*P. persica* is cultivated worldwide for its fruit, namely peaches, and its flower has traditionally been utilized as a diuretic, purgative, and cosmetic in China and Korea; moreover, it is currently used as a weight loss tea [8,10,11]. *P. persica* flowers mainly contain phenolic compounds and flavonoids, and their major components include quercetin derivatives, kaempferol and its derivatives, and chlorogenic acid [12,13,14,15]. Their pharmacological actions are mostly beauty-related, including applications such as anti-obesity [8], skin-whitening [16], and the prevention of ultraviolet-induced photoaging [12,13]. In addition, purgative and prokinetic actions have been reported [10,14]. *N. nucifera*, commonly called lotus, is an aquatic crop, and its leaves have been widely used in Asia as a tea for weight loss, a food wrapper for cooking, and a traditional medicine for the management of obesity, hyperglycemia, and hyperlipidemia [17,18]. The bioactive constituents of *N. nucifera* leaves are mainly alkaloids, such as nuciferine and nornuciferine, which are responsible for its various pharmacological activities, including anti-obesity, anti-hyperlipidemia, anti-inflammatory, anti-hyperuricemic, sedative-hypnotic, and anxiolytic effects [17,19,20,21]. Although these two herbs have been used to manage obesity and associated metabolic disorders, the mixture of the two has not been well studied, and its effective dose and possible mechanisms of action remain unknown.

The present study investigated the anti-obesity effects of an herbal extract mixture of *P. persica* and *N. nucifera* (designated as HT077) and explored the underlying mechanisms using an HFD-induced obesity model. Male C57BL/6 mice were fed an HFD mixed with 0.1%, 0.2%, or 0.4% HT077 for 12 weeks and their body weight, abdominal fat mass, spleen and liver weights, and serum biochemical profiles were assessed. We also measured hepatic triglyceride and total cholesterol contents and examined the expression levels of genes related to lipogenesis and fatty acid oxidation in adipose tissues.

## 2. Materials and Methods

### 2.1. Sample Preparation

HT077 is a mixture of water extracts of *P. persica* flowers and *N. nucifera* leaves. Dried *P. persica* flowers and *N. nucifera* leaves were purchased from Hebei Cao Mu Trade Co., Ltd. (Hebei, China) and Young Chang Medicinal Herbs Co. (Seoul, Korea), respectively, and were authenticated by Professor Hocheol Kim of the College of Korean Medicine, Kyung Hee University. *P. persica* was first extracted for 2 h with 20 volumes of distilled water at 100 °C and then extracted for 2 h with 15 volumes of distilled water at 100 °C. *N. nucifera* was extracted for 3 h with 30 volumes of distilled water at 100 °C. *P. persica* and *N. nucifera* extracts were individually cooled at room temperature, filtered, and concentrated under reduced pressure to 30 and 20 brix, respectively. Concentrated extracts were individually mixed with dextrin and spray-dried to yield powders (extraction yields of 49% and 22% for *P. persica* and *N. nucifera*, respectively). The extract powders of *P. persica* and *N. nucifera* were mixed at a ratio of 57:43. Voucher specimens of the raw materials (no. 190,611,002 and 190,627,051) were deposited in the Herbarium of NeuMed Inc. (Seoul, Korea). Multiflorin B and nuciferine, the marker compounds of *P. persica* and *N. nucifera*, respectively [12,22], were quantified as 0.63 mg/g and 0.84 mg/g of dried HT077 extract, respectively, according to the high-performance liquid chromatography analysis.

### 2.2. Animals and Experimental Design

Three-week-old male C57BL/6 mice were purchased from Orient Bio Inc. (Seongnam-si, Gyeonggi-do, Korea) and acclimatized for 1 week before use. Mice were housed four per cage under controlled laboratory conditions of 23 ± 1 °C, 55% ± 5% humidity, and a 12 h light/dark cycle. After the acclimation period, the mice were randomly divided into six groups as follows: normal diet (ND), HFD control, HFD + orlistat (positive control), and HFD + 0.1, 0.2, or 0.4% HT077 (*n* = 8 for ND, 16 for HFD, and 12 for the other four groups). The ND group was fed a rodent diet with 10 kcal% fat (cat. no. D12450B; Research Diets Inc., New Brunswick, NJ, USA) for 12 weeks, while the other HFD groups were fed a rodent diet with 60 kcal% fat (cat. no. D12492; Research Diets Inc.) with or without interventions. The positive control group received an HFD mixed with 0.02% orlistat, and the HFD + HT077 groups received an HFD mixed with 0.1, 0.2, or 0.4% HT077 extract. During the experimental period, mice were allowed ad libitum access to diet and distilled water. Mice were checked daily for clinical signs including general appearance, posture, consciousness, behavior, breathing, salivation, vomiting, and so forth, and body weight and food intake were measured twice per week. At the end of 12 weeks, the mice were fasted overnight and sacrificed under isoflurane anesthesia. Blood was collected from the inferior vena cava, and epididymal, perirenal, and mesenteric adipose tissues, liver, and spleen were dissected and weighed. The adipose tissues were identified and isolated according to Bagchi and MacDougald [23] for accurate and reproducible measurement. The relative organ weights were calculated on the basis of the fasted body weight. The blood samples were let stand for 30 min at room temperature and centrifuged at 3000 rpm at 4 °C for 10 min to separate the serum. The adipose tissues, liver, and serum were stored at −80 °C before analysis. All experimental protocols were approved by the International Animal Care and Use Committee of the Korea Institute of Science and Technology for Eastern Medicine (approval no. KISTEM-IACUC-2019-004).

### 2.3. Serum Biochemical Analysis

Glucose, lipid, liver enzyme, and adipokine profiles were evaluated in serum. The levels of glucose, triglycerides, total cholesterol, aspartate aminotransferase (AST), and alanine aminotransferase (ALT) were analyzed using an automated clinical chemistry analyzer (DRI-CHEM NX700, Fujifilm Corp., Tokyo, Japan) according to the manufacturer’s instructions. The levels of leptin and adiponectin were determined using Quantikine^®^ ELISA kits (catalog no. MOB00 and MRP300, respectively; R&D Systems, Inc., MN, USA) according to the manufacturer’s instructions.

### 2.4. Determination of Hepatic Lipid Content

Lipids were extracted according to the method of Folch et al. [24] with slight modifications. Briefly, liver tissue (100 mg) was homogenized in 800 µL of methanol–chloroform solution (1:2, *v/v*) and 200 µL of distilled water. The hepatic mixture was vortexed and centrifuged at 12,000 rpm for 10 min. The lower phase of the mixture was separated and dried using nitrogen gas. The concentrations of triglyceride and total cholesterol in the liver were analyzed using commercial kits (catalog no. AM157S and AM202, respectively; Asan Pharmaceutical, Seoul, Korea).

### 2.5. Real-Time Polymerase Chain Reaction (PCR) Analysis

Total RNA was extracted from mesenteric adipose tissue using QIAzol lysis reagent (cat. no. 79306; Qiazen, Venlo, Netherlands) and reverse-transcribed to complementary DNA using a commercial reverse transcription kit (cat. no. 4368814; Applied Biosystems, Waltham, MA, USA) according to the manufacturer’s instructions. PCR amplifications were performed on a StepOnePlus™ Real-Time PCR System (Applied Biosystems Inc., Bedford, MA, USA) using an SYBR Green PCR Master Mix (cat. no. 4367659; Applied Biosystems Inc., Bedford, MA, USA). The sequences of the sense and antisense primers were as follows: 5′-ACAGTCCAGCCTTTGAGGATAG-3′ and ′-GACACAGAAAGGCCAGTACACA-3′ for sterol regulatory element-binding protein-1c (*SREBP-1c*); 5′-GGCAGCAGTTACACCACATAC-3′ and 5′-TCATTACCTCAATCTCAGCATAGC-3′ for acetyl-CoA carboxylase (*ACC*); 5′-GTACAGGCTGAAGGAGGACACT-3′ and 5′-TGAGATGTGGATACCACCAGAG-3′ for fatty acid synthase (*FAS*); 5′-CTGACCTACTACTTCAAGGGCAGT-3′ and 5′-GGGAGTCTGTATGAATACCTCTGC-3′ for stearoyl-CoA desaturase-1 (*SCD-1*); 5′-AGGAGCTCCAAGACTCTAGACAAC-3′ and 5′-CAAAGTCTCTCTCAGGTAGCACTG-3′ for peroxisome proliferator-activated receptor-γ coactivator-1α (*PGC-1α*); 5′-CAGTGGGGAGAGAGGACAGA-3′ and 5′-AGTTCGGGAACAAGACGTTG-3′ for peroxisome proliferator-activated receptor α (*PPARα*); 5′-CCAGGCTACAGTGGGACATT-3′ and 5′-GAACTTGCCCATGTCCTTGT-3′ for carnitine palmitoyltransferase-1 (*CPT-1*); 5′-ACAATGAATACGGCTACAGCAACAG-3′ and 5′-GGTGGTCCAGGGTTTCTTACTCC-3′ for glyceraldehyde-3-phosphate dehydrogenase. The relative gene expression levels were calculated by the 2^−ΔΔCt^ method.

### 2.6. Statistical Analysis

Values are expressed as the mean ± standard deviation (SD). One-way analysis of variance with Dunnett’s post hoc test was performed to compare more than two groups, whereas independent t-tests were performed to compare two groups. All analyses were performed using Prism 5 software (GraphPad Software, Inc., San Diego, CA, USA). Statistical significance was set at *p* < 0.05.

## 3. Results

### 3.1. Effects of HT077 on Body Weight and Food Consumption

**Figure 1:**
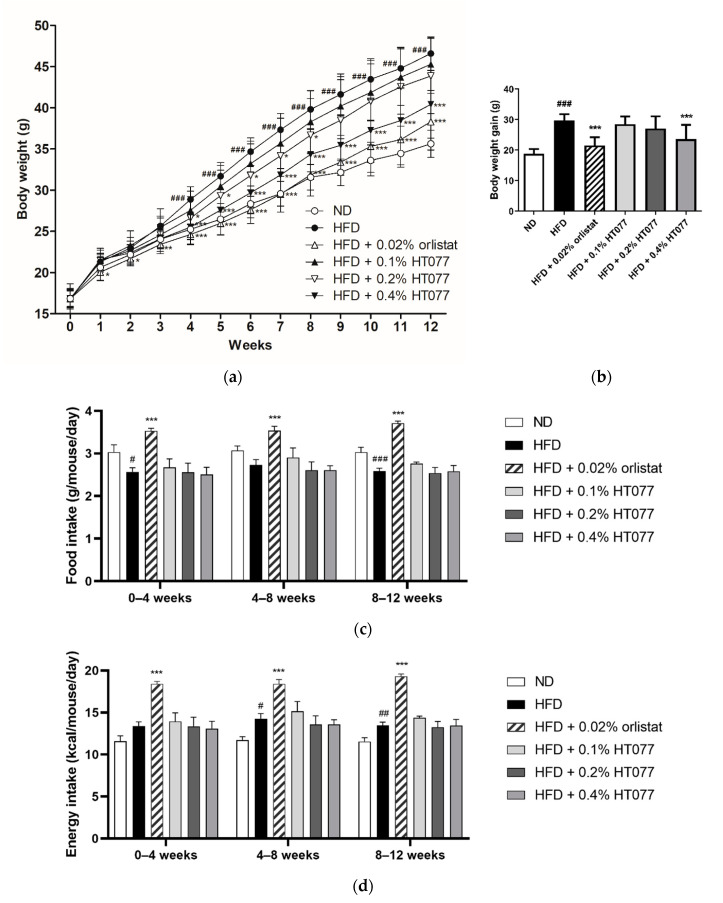
Effects of an extract mixture of *Prunus persica* and *Nelumbo nucifera* (HT077) on body weight, and food and energy consumption in C57BL/6 mice fed a high-fat diet (HFD). (**a**) Body weight changes in mice that received either a normal diet (ND), HFD, HFD containing 0.02% orlistat, or HFD containing 0.1, 0.2, or 0.4% HT077 for 12 weeks. (**b**) Body weight gain for 12 weeks. Changes in (**c**) food intake and (**d**) energy intake over 12 weeks. All values are presented as the mean ± SD (*n* = 8 for ND, 16 for HFD, and 12 for the other groups). ^#^ *p* < 0.05, ^##^ *p* < 0.01, and ^###^ *p* < 0.001 vs. ND group; * *p* < 0.05, ** *p* < 0.01, and *** *p* < 0.001 vs. HFD control group.

The body weights of animals in the HFD control group began to increase significantly compared with those in the ND group at week 4, and the difference between the two groups grew until the end of the study period (Figure 1a). Supplementation with 0.2% HT077 significantly reduced body weight at 4–8 weeks and tended to reduce body weight at 9–12 weeks (not significant) compared with the HFD control group. Supplementation with 0.4% HT077 and 0.02% orlistat significantly decreased body weights at 4–12 and 1–12 weeks, respectively, compared with those in the HFD control group. At the end of the experiment, mice fed an HFD containing 0.4% HT077 showed markedly reduced body weight gains compared with those in the HFD control mice (both *p <* 0.001, Figure 1b). HFD-fed control mice showed significantly lower food intake (weeks 0–4 and 8–12) or lower food intake (weeks 4–8) than ND-fed mice during the study period (Figure 1c). HFD-fed control mice had significantly higher energy intake (weeks 4–12) or higher energy intake (weeks 0–4) than ND-fed mice (Figure 1d). There was no significant difference in food intake and energy intake between the HFD + HT077 and HFD control groups. The food and energy intakes of the HFD + 0.02% orlistat group were significantly higher than those of the HFD control group for 12 weeks. In addition, no abnormal clinical signs of toxicity were observed in the HFD + HT077 group during the experimental period.

### 3.2. Effects of HT077 on Abdominal Fat, Liver, and Spleen Wet Weights

**Figure 2:**
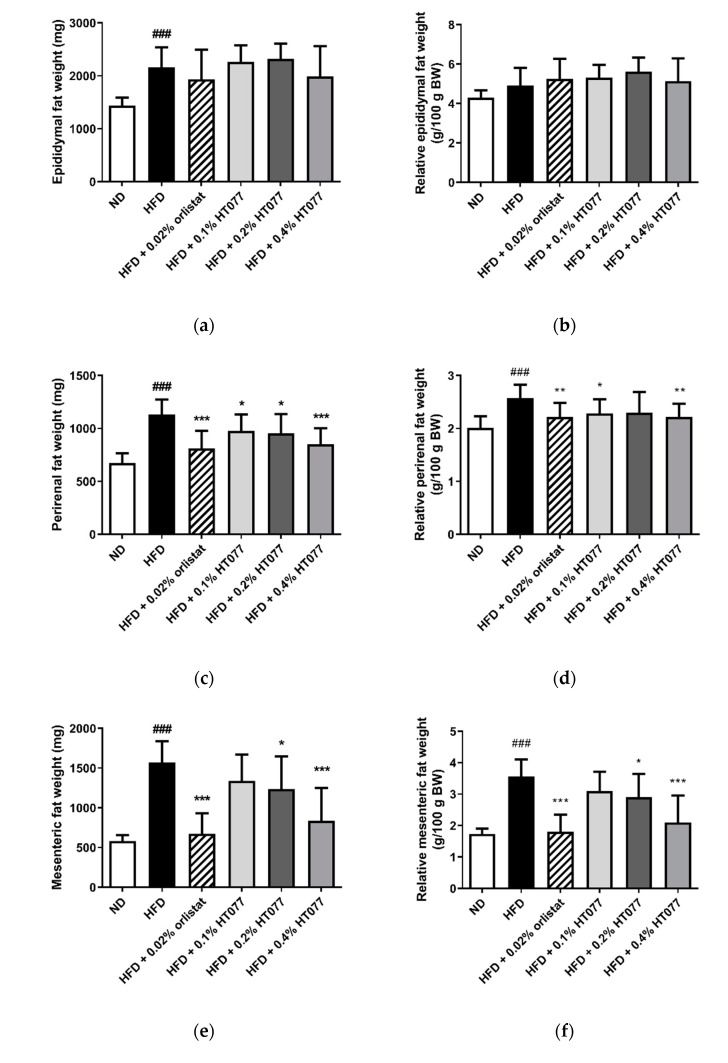

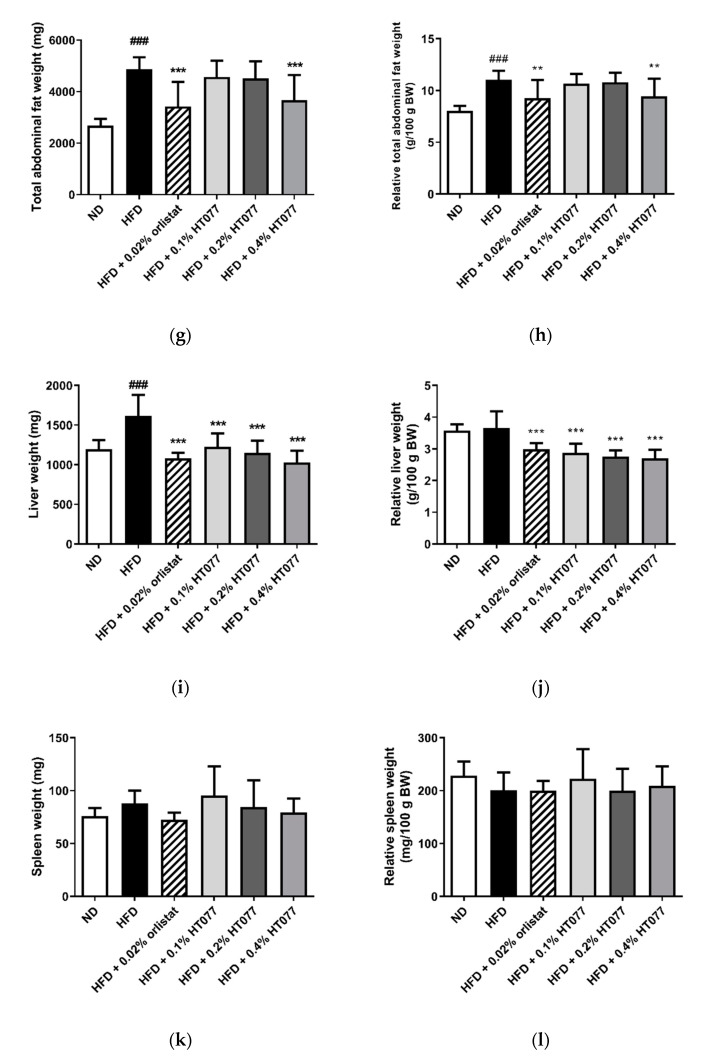
Effects of an extract mixture of *Prunus persica* and *Nelumbo nucifera* (HT077) on the absolute and relative weights of abdominal fat (**a–h**), liver (**i–j**), and spleen (**k–l**) in C57BL/6 mice fed a high-fat diet (HFD). The absolute wet weights of white adipose tissues isolated from visceral regions including epididymal (**a**), perirenal (**c**), and mesenteric (**e**) deposits were measured. Total abdominal fat weights (**g**) are the sums of three individual fat depots (**a**,**c**,**e**). The relative organ weight was calculated with respect to fasted body weight. Mice received either a normal diet (ND), HFD, HFD containing 0.02% orlistat, or HFD containing 0.1, 0.2, or 0.4% HT077 for 12 weeks. All values are presented as the mean ± SD (*n* = 8 for ND, 16 for HFD, and 12 for the other groups). ^###^ *p <* 0.001 vs. ND group; * *p* < 0.05 and ** *p* < 0.01, *** *p* < 0.001 vs. HFD control group. BW, body weight.

The absolute and relative weights of total abdominal, perirenal, and mesenteric adipose tissue and the absolute epididymal adipose tissue weight were significantly increased in the HFD control group compared with those in the ND group, showing that 12 weeks of HFD feeding caused obesity (all *p <* 0.001, Figure 2a–h). In contrast, supplementation with HT077 and orlistat significantly reduced the absolute and relative weights of perirenal, mesenteric, and total abdominal fat pad compared with those in the HFD control group (Figure 2c–h). HFD feeding increased the absolute liver weights compared with the those in the ND group (*p <* 0.001, Figure 2i), whereas supplementation with 0.02% orlistat and 0.1%, 0.2%, and 0.4% HT077 significantly reduced the absolute and relative liver weights compared with those in the HFD group (all *p <* 0.001, Figure 2i,j). No difference was observed in spleen weights among the groups (Figure 2k,l).

### 3.3. Effects of HT077 on Serum Levels of Glucose, Lipids, and Liver Enzymes

**Figure 3:**
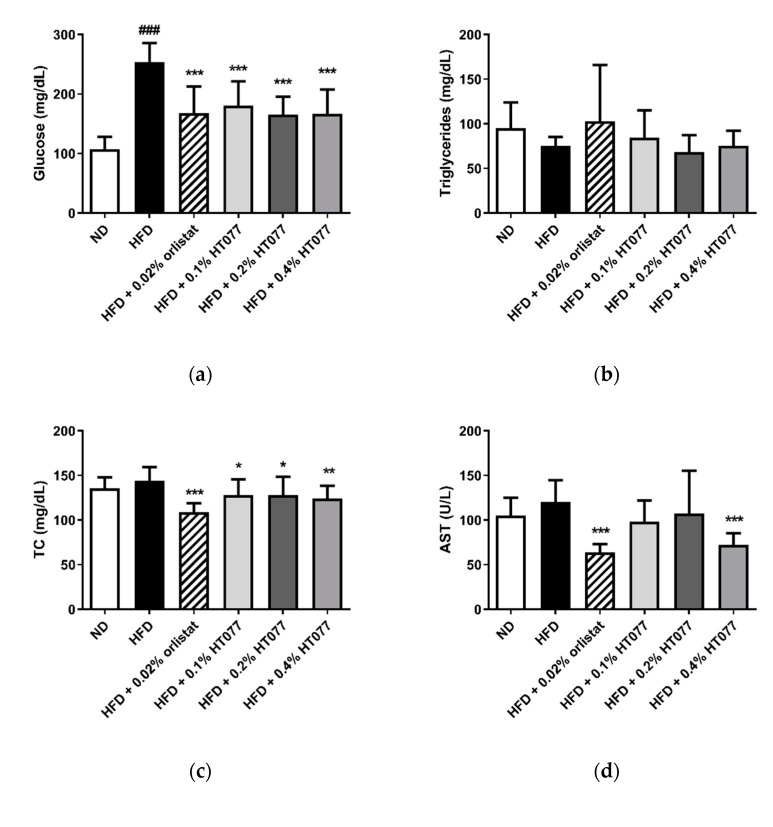

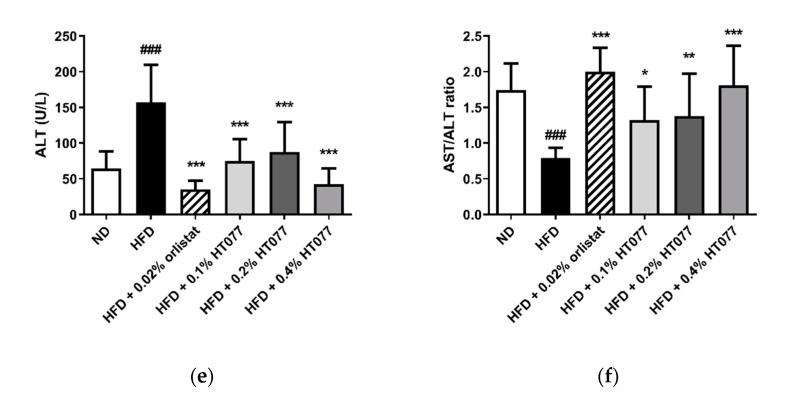
Effects of an extract mixture of *Prunus persica* and *Nelumbo nucifera* (HT077) on serum biochemical profiles in C57BL/6 mice fed a high-fat diet (HFD). The levels of glucose (**a**), triglycerides (**b**), total cholesterol (**c**), AST (**d**), and ALT (**e**), as well as the AST/ALT ratio (**f**), were measured. Mice received either a normal diet (ND), HFD, HFD containing 0.02% orlistat, or HFD containing 0.1, 0.2, or 0.4% HT077 for 12 weeks. All values are presented as the mean ± SD (*n* = 8 for ND, 16 for HFD, and 12 for the other groups). ^###^ *p* < 0.001 vs. ND group; * *p* < 0.05, ** *p* < 0.01, and *** *p* < 0.001 vs. HFD control group. ALT, alanine aminotransferase; AST, aspartate aminotransferase; TC, total cholesterol.

HFD feeding significantly increased serum glucose levels compared with those in the ND group (*p* < 0.001, Figure 3a). In contrast, supplementation with 0.02% orlistat and 0.1%, 0.2%, and 0.4% HT077 significantly reduced glucose levels compared with those in the HFD control group (all *p* < 0.001). No significant differences were observed in triglyceride levels among the groups (Figure 3b). Total cholesterol levels were not altered by HFD feeding but were significantly reduced by supplementation with 0.02% orlistat and 0.1%, 0.2%, and 0.4% HT077 compared with those in the HFD control group (Figure 3c). Likewise, serum AST levels were not altered by HFD feeding but were significantly reduced by supplementation with 0.02% orlistat and 0.4% HT077 compared with those in the HFD control group (both *p* < 0.001, Figure 3d). The HFD significantly elevated serum ALT levels and decreased the AST/ALT ratio compared with those in the ND group (both *p* < 0.001, Figure 3e,f), whereas supplementation with 0.02% orlistat and 0.1%, 0.2%, and 0.4% HT077 reversed these changes.

### 3.4. Effects of HT077on Serum Adipokine Concentrations

**Figure 4:**
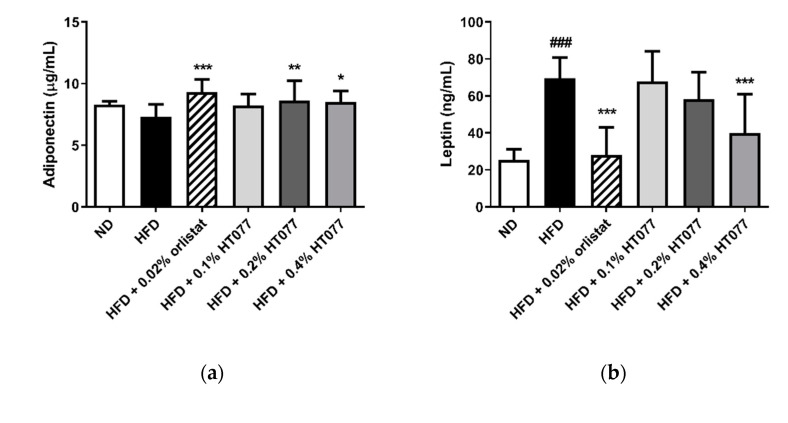

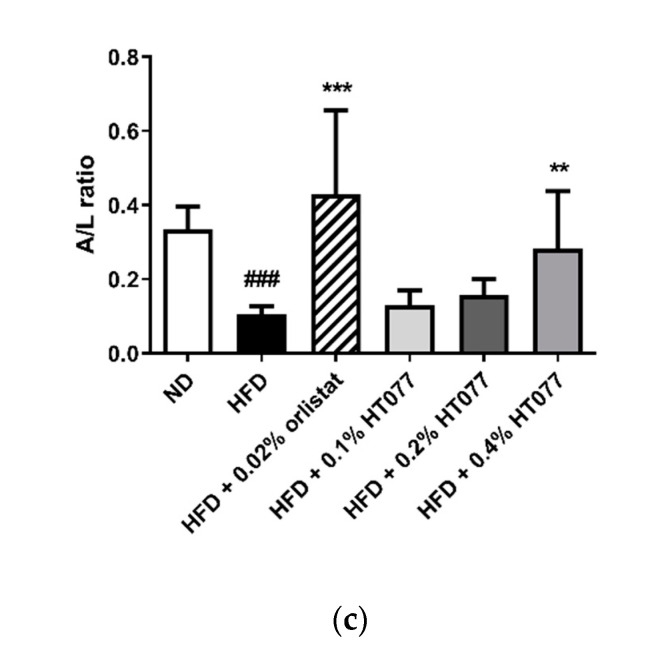
Effects of an extract mixture of *Prunus persica* and *Nelumbo nucifera* (HT077) on serum adiponectin (**a**) and leptin (**b**) levels and the adiponectin/leptin (A/L) ratio (**c**) in C57BL/6 mice fed a high-fat diet (HFD). Mice received either a normal diet (ND), HFD, HFD containing 0.02% orlistat, or HFD containing 0.1, 0.2, or 0.4% HT077 for 12 weeks. All values are presented as the mean ± SD (*n* = 8 for ND, 16 for HFD, and 12 for the other groups). ^###^ *p* < 0.001 vs. ND group; * *p* < 0.05, ** *p* < 0.01, and *** *p* < 0.001 vs. HFD control group.

Adiponectin levels tended to decrease after HFD feeding (statistically not significant), but significantly increased with 0.02% orlistat and 0.2% and 0.4% HT077 supplementation, as compared with levels in the HFD control group (Figure 4a). HFD feeding significantly increased serum leptin levels and decreased the adiponectin/leptin ratio compared with those in the ND group (*p* < 0.001, Figure 4b, c), whereas supplementation with 0.02% orlistat and 0.4% HT077 significantly reversed these changes.

### 3.5. Effects of HT077on Hepatic Triglyceride and Total Cholesterol Contents

**Figure 5:**
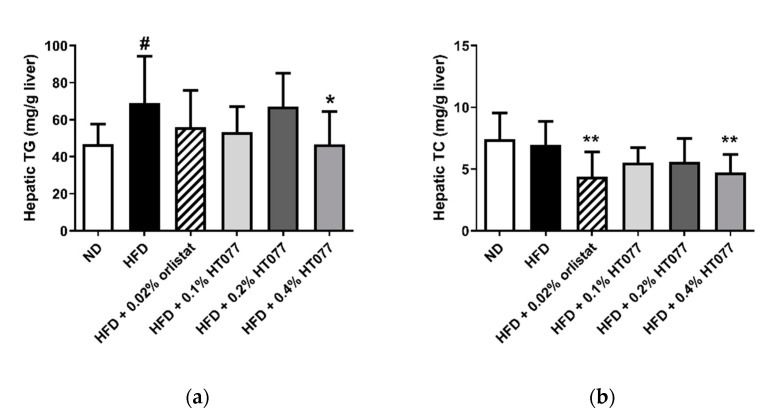
Effects of an extract mixture of *Prunus persica* and *Nelumbo nucifera* (HT077) on hepatic triglyceride (**a**) and total cholesterol (**b**) contents in C57BL/6 mice fed a high-fat diet (HFD). Mice received either a normal diet (ND), HFD, HFD containing 0.02% orlistat, or HFD containing 0.1, 0.2, or 0.4 HT077 for 12 weeks. All values are presented as the mean ± SD (*n* = 8 for ND, 16 for HFD, and 12 for the other groups). ^#^ *p* < 0.05 vs. ND group. * *p* < 0.05 and ** *p* < 0.01 vs. HFD control group. TC, total cholesterol; TG, triglyceride.

Twelve weeks of HFD feeding significantly increased triglyceride levels in the liver (*p* < 0.05, Figure 5a) but did not change total cholesterol levels (Figure 5b). Hepatic triglyceride levels were significantly reduced by supplementation with 0.4% HT077 (*p* < 0.05) but not 0.02% orlistat. Hepatic total cholesterol levels were significantly decreased by supplementation with 0.4% HT077 and 0.02% orlistat (both *p* < 0.01).

### 3.6. Effects of HT077on Lipogenesis and Fatty Acid Oxidation-Related Gene Expression in the Adipose Tissue

**Figure 6:**
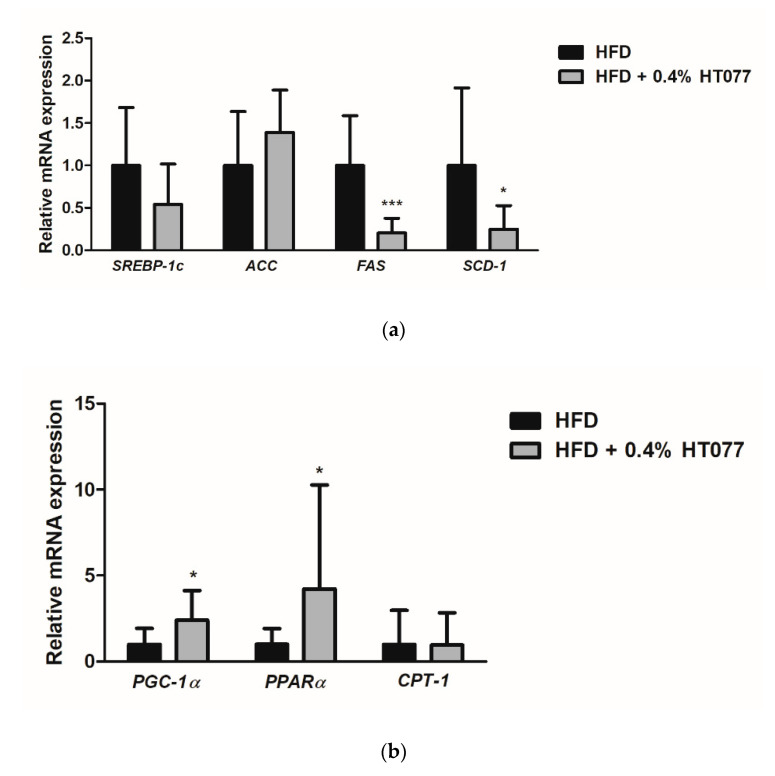
Effects of an extract mixture of *Prunus persica* and *Nelumbo nucifera* (HT077) on the expression of genes related to lipogenesis (**a**) and fatty acid oxidation (**b**) in the adipose tissue. C57BL/6 mice received a high-fat diet (HFD) or HFD containing 0.4% HT077 for 12 weeks. The mRNA expression levels of lipogenesis-related genes, including sterol regulatory element-binding protein 1c (*SREBP-1c*), acetyl-CoA carboxylase (*ACC*), fatty acid synthase (*FAS*), and stearoyl-CoA desaturase-1 (*SCD-1*), as well as fatty acid oxidation-related genes, including peroxisome proliferator-activated receptor-γ coactivator-1α (*PGC-1α*), peroxisome proliferator-activated receptor α (*PPARα*), and carnitine palmitoyltransferase-1 (*CPT-1*), were measured in the mesenteric white adipose tissue. All values are presented as the mean ± SD (*n* = 16 for HFD and 12 for HFD + HT077 groups). The *y*-axis indicates relative gene expression normalized to that in the control group. * *p* < 0.05 and *** *p* < 0.001 vs. HFD control group.

To explore the possible mechanisms underlying the observed effects of HT077, gene expression related to lipid metabolism in mesenteric adipose tissue was compared between HFD and HFD + HT077 groups. Since a high dose (0.4%) was the most effective at reducing adiposity, the HFD + 0.4% HT077 group was chosen for analysis. HT077 tended to decrease the expression levels of *SREBP-1c* in mesenteric adipose tissue compared with those in the HFD control mice (*p* = 0.0574, Figure 6a). The mRNA levels of *FAS* and *SCD-1* were significantly decreased in the HFD + 0.4% HT077 group compared with those in the HFD control group (*p* < 0.001 and *p* < 0.01, respectively), whereas there was no difference in *ACC* mRNA levels between the two groups. The mRNA levels of *PGC-1a* and *PPARα* were significantly increased in the HFD + 0.4% HT077 group compared with those in the HFD control group (both *p* < 0.05, Figure 6b), whereas *CPT1* mRNA levels were not different between the two groups.

## 4. Discussion

This study revealed that the herbal mixture of *P. persica* and *N. nucifera*, which are commonly used as teas for weight loss in East Asia, reduced body weight and fat accumulation in the adipose tissue and livers of obese mice. In addition, HT077 improved circulating glucose, lipid, and adipokine profiles and regulated the expression of genes involved in lipid metabolism in adipose tissue.

HT077 reduced body weight and visceral fat in HFD-fed mice without noticeable changes in food and energy consumption. HFD-fed rodents had increased caloric consumption and decreased food intake compared with ND-fed rodents, which is possibly due to impaired satiation signaling, and leads to fat accumulation and weight gain [25]. It is well established that visceral fat is the main contributor to abdominal obesity and is related to an increased risk of metabolic dysfunction. Representative visceral fat deposits in rodents include those of the epididymal, perirenal, and mesenteric fat pad [26]. Among these, the accumulation of perirenal fat increases sodium reabsorption due to kidney compression and activates the renin–angiotensin–aldosterone system, causing the development of hypertension, insulin resistance, and atherosclerosis [27,28,29]. Mesenteric fat drained through portal circulation has higher lipolysis activities than nonportal adipose tissue, and its excessive accumulation is expected to increase free fatty acid flux to the liver, leading to fatty liver and insulin resistance [30,31]. Notably, HT077 at a high dose (0.4%) reduced the perirenal and mesenteric fat weights by 61.4 and 74.4%, respectively, compared with those in the control group. HT077 did not affect food and energy consumption, whereas orlistat, a lipase inhibitor, increased both parameters. In line with our results, intestinal lipase inhibition has been shown to increase appetite and food consumption, which may counteract the therapeutic effects of orlistat [32]. Our results indicate that HT077 exerts promising anti-obesity effects in the absence of a change in appetite.

Long-term HFD intake causes ectopic fat deposition in several organs other than adipose tissue, such as liver and spleen. HFD feeding for 12 weeks induced a significant increase in liver weight but not in spleen weight, which is consistent with previous results [33]. HT077 inhibited hepatic fat accumulation and liver weight gain in HFD-fed mice, along with decreased ALT and AST levels and an increased AST/ALT ratio. Hepatic fat accumulation arises from increased circulating fatty acid uptake and decreased β-oxidation in the liver, leading to NAFLD [34]. Furthermore, increased hepatic fat is known as an independent indicator of obesity-related metabolic complications [35]. NAFLD following HFD is denoted by the elevated leakage of liver enzymes from hepatocytes into the blood, with that of ALT being more prominent than that of AST, and by an AST/ALT ratio less than 1 [36,37], all of which are consistent with our results. *P. persica* and *N. nucifera* are known to inhibit fatty liver induced by HFD in animals by regulating hepatic lipogenesis [8,38,39,40]. Together with these previous findings, our results suggest that HT077 might prevent obesity-related fatty liver diseases.

Obesity is commonly a co-morbidity of glucose and lipid metabolism disorders. HFD consumption increases hepatic glucose output and decreases glucose uptake in peripheral tissues, resulting in hyperglycemia [41]. This increases insulin secretion and consequently activates hepatic lipogenesis and cholesterol excretion, leading to hyperlipidemia [41]. In this study, the serum levels of glucose and total cholesterol were significantly reduced after HT077 supplementation, indicating hypoglycemic and hypocholesterolemic activities. Previous studies have shown that *P. persica* and its major component multiflorin A reduce blood glucose levels in HFD-fed and glucose-loaded mice, respectively [8,42]. Further, *N. nucifera* improves glucose tolerance and insulin resistance in rodents fed an HFD [43,44] and shows anti-hyperglycemic activity in diabetic rats [45]. Moreover, we previously reported the synergistic effects of combined *P. persica* and *N. nucifera* on hypoglycemic activity in HFD-induced obese mice [9]. In addition, *N. nucifera* and its total flavonoids are known to improve lipid profiles of rats fed an HFD [18,46]. Our results, along with those of previous studies, suggest that HT077 might improve obesity-associated hyperglycemia and hypercholesterolemia.

Over the past few decades, it has been established that adipose tissue is not simply an energy reservoir but is an endocrine organ that secretes adipokines such as leptin and adiponectin. Leptin induces weight and fat loss by suppressing appetite and elevating energy expenditure through action in the hypothalamus [47]. Leptin resistance, marked by hyperleptinemia, occurs universally in human obesity and is a key feature of HFD-induced obesity in rodents [48]. It is known to exacerbate adiposity and hepatic triglyceride accumulation and impair the homeostasis of peripheral glucose and lipid metabolism [49]. Adiponectin has been shown to regulate peripheral glucose and fatty acid metabolism by increasing the insulin sensitivity of target organs, including the liver and muscle [50]. In obesity and metabolic syndrome, circulating adiponectin levels decrease and contribute to the development of insulin resistance [50]. In addition, the adiponectin/leptin ratio has recently been proposed as an indicator of adipose tissue dysfunction [51]. An increase in this ratio is better correlated with reduced insulin resistance than leptin or adiponectin alone [52]. Herein, HT077 significantly decreased circulating leptin levels and increased adiponectin levels and the adiponectin/leptin ratio. In support of our results, nuciferine, a major active alkaloid in *N. nucifera* leaf, reportedly reduces leptin and increases adiponectin levels in HFD-fed hamsters [53]. Our results indicate that improvements in leptin and adiponectin levels following HT077 administration might contribute to counteracting HFD-induced excessive lipid accumulation and abnormal glucose and lipid metabolism.

Adipose tissue plays an important role in the regulation of lipid metabolism and its metabolic functions include lipogenesis and fatty acid oxidation [54]. To better understand the mechanisms underlying the effects of HT077, we analyzed gene expression related to lipid metabolism in mesenteric adipose tissue. Mesenteric fat is known to be the most similar visceral tissue between humans and rodents because of its high vascularity and portal venous drainage [55,56], and in this study, was the site where lipid accumulation was most inhibited by HT077. In C57BL/6 mice, HFD affects lipid metabolism in adipose tissue differently depending on the site of adipose tissue [57,58]. In mesenteric adipose tissue, lipogenic genes such as *SREBP-1c*, *ACC*, *FAS*, and *SCD-1* are upregulated by an HFD compared with a ND, and inhibition of this upregulation exerts anti-obesity effects [58,59,60,61]. The expression of *FAS* and *SCD-1* was significantly decreased by HT077 administration compared with that in the control mice. FAS and SCD-1 are central enzymes in *de novo* lipogenesis in adipose tissue [62]. FAS catalyzes the biosynthesis of saturated long-chain fatty acids from acetyl and malonyl coenzyme A and NADPH, whereas SCD-1 catalyzes the biosynthesis of monounsaturated fatty acids from their saturated fatty acid precursors [62]. Given that increased expression and activity of FAS and SCD-1 in adipose tissue contribute to the development of obesity and insulin resistance [63,64], decreased lipid synthesis through the inhibition of FAS and SCD-1 expression might contribute to the inhibitory effects of HT077 on adiposity and related metabolic disorders. Furthermore, an HFD upregulates the expression of fatty acid oxidation-related genes such as *PGC-1α*, *PPARα*, and *CPT-1* in C57BL/6 mice and this upregulation attenuates fat accumulation in adipose tissue and liver [57,58]. HT077 increased the expression of *PGC-1α* and *PPARα* compared with that in the control mice. PGC-1α and PPAR-α are key regulators of mitochondrial biogenesis. Their overexpression in adipocytes induces the expression of target genes involved in fatty acid oxidation, thereby inhibiting fat accumulation and improving insulin resistance [65,66]. Increased fatty acid oxidation in mesenteric fat induced by HT077 might contribute to the inhibition of free fatty acid influx into the liver, suppressing the development of fatty liver. Overall, our results suggest that the anti-obesity effects of HT077 are at least partially related to decreased lipogenesis and increased fatty acid oxidation in adipose tissue.

HT077 showed dose-dependent effects in reducing abdominal and hepatic fat accumulation and improving adipokine imbalances. Notably, HT077 at a high dose (0.4%) inhibited fat accumulation similarly to orlistat. Glucose and total cholesterol levels were lowered similarly by all doses of HT077, and in particular, the hypoglycemic efficacy was comparable to that of orlistat. Our results overall suggest that high-dose HT077 therapy may provide beneficial effects as orlistat does. When calculated based on the food intake and body weight, the average daily doses of 0.1, 0.2, and 0.4% HT077 groups are approximately 90, 180, and 360 mg/kg of mouse body weight, respectively, and that of 0.02% orlistat group is 25 mg/kg. The mouse dose of orlistat corresponds to a human equivalent dose of about 120 mg/day for 60 kg person based on body surface area [67]. Considering that 90–180 mg/day dosage of orlistat resulted in an 8.5–8.8% reduction of initial body weight in a 6-month dose-ranging study of obese subjects [68], HT077 has a favorable anti-obesity efficacy.

Given that HT077 decreased *FAS* and *SCD-1* expression and increased *PGC-1α* and *PPARα* expression in white adipose tissue (WAT), it is tempting to speculate that HT077 prevents HFD-induced weight gain by increasing energy expenditure via induction of WAT browning. Previous studies have shown that targeted deletion of *FAS* in adipose tissue induces brown fat-like adipocytes in WAT, increases energy expenditure, and decreases diet-induced obesity [69]. SCD-1 deficiency potentiates beige adipocyte formation and increases energy expenditure in HFD-fed mice [70]. In addition, overexpression of PGC-1α and PPARα induces brown fat feature in white adipocytes [71]. As browning of white fat significantly contributes to an increase in whole-body energy expenditure [72], HT077 is hypothesized to increase energy expenditure and this hypothesis requires further investigation to measure the energy expenditure of animals using metabolic cages, which was not examined in this study. In addition, the effects of HT077 on obesity-induced insulin resistance could be investigated since *Nelumbo nucifera* and its active constituent nuciferine have been shown to decrease serum insulin levels and homeostasis model assessment for insulin resistance index in HFD-induced obese rodents [44,53].

## 5. Conclusions

Our results suggest that HT077 has anti-obesity effects and inhibits the development of obesity-related metabolic disorders such as fatty liver, hyperglycemia, and hyperlipidemia. These effects might be partially mediated by an improvement in blood leptin and adiponectin concentrations and the regulation of lipid synthesis and fatty acid oxidation in adipose tissue. Our results support the potential health benefits of HT077 in managing obesity and related metabolic diseases.
